# *Biomphalaria glabrata* Metallothionein: Lacking Metal Specificity of the Protein and Missing Gene Upregulation Suggest Metal Sequestration by Exchange Instead of through Selective Binding

**DOI:** 10.3390/ijms18071457

**Published:** 2017-07-06

**Authors:** Michael Niederwanger, Sara Calatayud, Oliver Zerbe, Sílvia Atrian, Ricard Albalat, Mercè Capdevila, Òscar Palacios, Reinhard Dallinger

**Affiliations:** 1 Institute of Zoology and Center of Molecular Biosciences Innsbruck (CMBI), University of Innsbruck, Technikerstraße 25, A-6020 Innsbruck, Austria; michael.niederwanger@uibk.ac.at; 2 Departament de Genètica, Microbiologia i Estadística and Institut de Recerca de la Biodiversitat (IRBio), Universitat de Barcelona, Av. Diagonal 643, E-08028 Barcelona, Spain; scalatro7@alumnes.ub.edu (S.C.); ralbalat@ub.edu (R.A.); 3 Department of Chemistry, University of Zürich, Winterthurerstrasse 190, CH-8057 Zürich, Switzerland; oliver.zerbe@chem.uzh.ch; 4 Departament de Química, Facultat de Ciències, Universitat Autònoma de Barcelona, E-08193 Cerdanyola del Vallès, Spain; merce.capdevila@uab.cat (M.C.); oscar.palacios@uab.cat (Ò.P.)

**Keywords:** metallothionein, metal-binding, zinc, copper, cadmium, *Biomphalaria glabrata*, *Gastropoda*, *Hygrophila*

## Abstract

The wild-type metallothionein (MT) of the freshwater snail *Biomphalaria glabrata* and a natural allelic mutant of it in which a lysine residue was replaced by an asparagine residue, were recombinantly expressed and analyzed for their metal-binding features with respect to Cd^2+^, Zn^2+^ and Cu^+^, applying spectroscopic and mass-spectrometric methods. In addition, the upregulation of the *Biomphalaria glabrata* *MT* gene was assessed by quantitative real-time detection PCR. The two recombinant proteins revealed to be very similar in most of their metal binding features. They lacked a clear metal-binding preference for any of the three metal ions assayed—which, to this degree, is clearly unprecedented in the world of *Gastropoda* MTs. There were, however, slight differences in copper-binding abilities between the two allelic variants. Overall, the missing metal specificity of the two recombinant MTs goes hand in hand with lacking upregulation of the respective *MT* gene. This suggests that in vivo, the *Biomphalaria glabrata* MT may be more important for metal replacement reactions through a constitutively abundant form, rather than for metal sequestration by high binding specificity. There are indications that the MT of *Biomphalaria glabrata* may share its unspecific features with MTs from other freshwater snails of the *Hygrophila* family.

## 1. Introduction

In many species of *Gastropoda* (snails and slugs), cadmium (Cd) and copper (Cu) metabolism and detoxification are apparently linked to the expression of metal-specific metallothionein (MT) isoforms. Terrestrial snails like the Roman snail (*Helix pomatia*) of the *Stylommatophora* phylum, for example, possess Cd and Cu-specific *MT* genes whose transcriptional activation can be induced by metal exposure, leading to the expression of two-domain MT proteins which preferentially bind and inactivate the respective cognate metal ion [1,2,3]. Likewise, snails of the taxonomic clade of *Caenogastropoda* such as the marine periwinkle (*Littorina littorea*), possess a Cd-specific MT whose expression is strongly induced by Cd exposure [4] and environmental stressors [5]. In contrast to terrestrial snails from the *Stylommatophora* phylum, however, the MTs of *Littorina littorea* and other species of the *Caenogastropoda* clade possess three metal-binding domains [4]. Overall, the metal-binding performance of most of these MTs can be attributed to an intact domain structure with an optimized metal-binding stoichiometry, where every domain provides nine cysteine-linked sulfur atoms that coordinate with high affinity three Cd^2+^ or four to six Cu^+^ ions. In accordance with the metal-specific binding preference of the expressed proteins, the respective *MT* genes can be upregulated in response to exposure to the corresponding metal ion [2,6]. It has been suggested that the high binding specificity of some snail MTs for Cd^2+^ may serve detoxification of this harmful metal by keeping Cd^2+^ pathways within the snail organism strictly separated from pathways of other, essential metal ions [1].

In freshwater pulmonate snails of the *Hygrophila* clade, the situation may be different. Although they possess MTs [7], an important pathway of metal detoxification in these species is represented by metal-binding to phytochelatins as shown, for example, for the Great Pond snail, *Lymnaea stagnalis* [8]. Accordingly, the capacity of *Hygrophila* to express *MT*s in response to metal stress is apparently inhibited or strongly reduced when compared to other gastropod species, as recently demonstrated in the Bladder snail, *Physa acuta* [9]. *Biomphalaria glabrata* is another species of *Hygrophila* that lives in tropical and subtropical rivers and ponds, being one of the most important intermediate hosts of the trematode, *Schistosoma mansoni* [10,11] that infects with schistosomiasis millions of people worldwide [12]. In the present study we have examined the metal-binding performance of two MT allelic variants of *Biomphalaria glabrata*, the wildtype form (BgwtMT) and a natural mutant in which a lysine residue has been replaced by an asparagine residue (BgKNMT). Last but not least, it was also explored how the *Biomphalaria glabrata* MT variants compare with structural and functional features of MTs from other *Hygrophila* species. In addition, the expression of the *Biomphalaria glabrata MT* gene was assessed under control and Cd exposure conditions.

## 2. Results and Discussion

### 2.1. The BgwtMT and BgKNMT Recombinant Proteins

**Figure 1:**
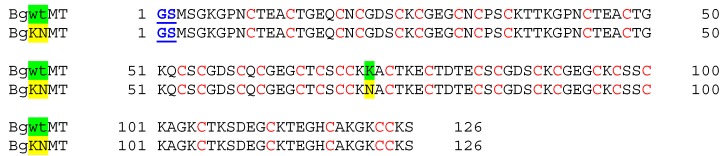
Amino acid sequences of the studied recombinant metallothioneins: Wild type (BgwtMT) and the naturally mutated (BgKNMT) proteins. **Red**: cysteine residues; underlined **blue**: N-terminal additional residues introduced due to the recombinant expression conditions (see material and methods section); shaded in **green**: wildtype position of K (BgwtMT); shaded in **yellow**: its replacement by N in the natural allelic mutant (BgNKMT). The two protein sequences can be found in the GenBank under the following accession numbers: KT697617 (BgwtMT); and KY963493 (BgKNMT).

The primary structure of the wildtype MT from *Biomphalaria glabrata* (BgwtMT) was originally obtained by translation from the nucleotide sequence of the corresponding gene (see below), and afterwards confirmed by mRNA isolation and sequencing from laboratory-grown living snails [13] (in preparation). The second sequence originates from a natural allelic MT variant with an amino acid replacement (K→N) (BgKNMT), and was chosen from a number of different allelic mutants, all of them characterized from individuals of the same laboratory-grown population. The primary structure of the two recombinant proteins is shown in Figure 1. Due to the specific recombinant expression conditions, the two proteins contain two additional amino acid residues (GS) at their N-termini in relation to their native isoforms previously characterized [13] (in preparation). As previously shown [14], these modifications do not interfere with the metal-binding capacity of the recombinant proteins.

**Figure 2:**
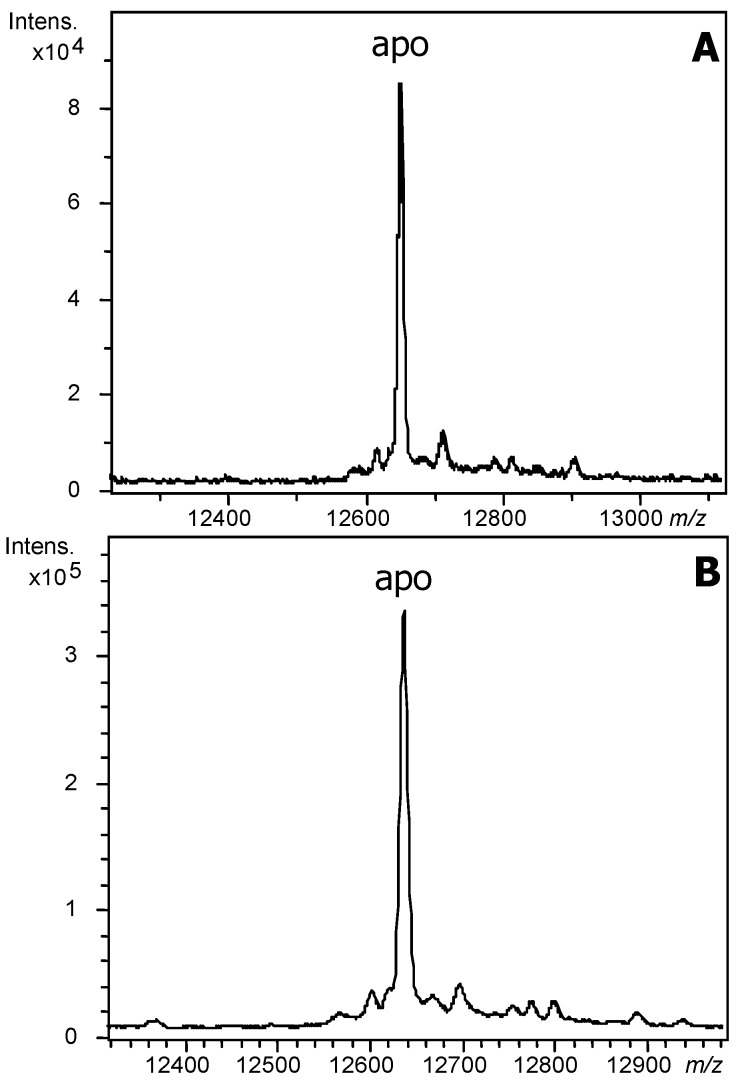
MS spectra of BgMT proteins: Deconvoluted electrospray ionization mass spectrometry (ESI-MS) spectra of the bacterial recombinant production of (**A**) BgwtMT (Experimental Molecular Mass: 12,652) and (**B**) BgKNMT (Experimental Mass: 12,639) in Zn-enriched media, recorded at pH 2.4.

The mass spectra of the recombinant productions of Zn-BgwtMT and Zn-BgKNMT recorded under acidic conditions (Figure 2) allowed confirmation of the expected masses of both proteins according to their sequences.

### 2.2. Divalent Zn(II) and Cd(II)-Binding Features of BgwtMT and BgKNMT

**Figure 3:**
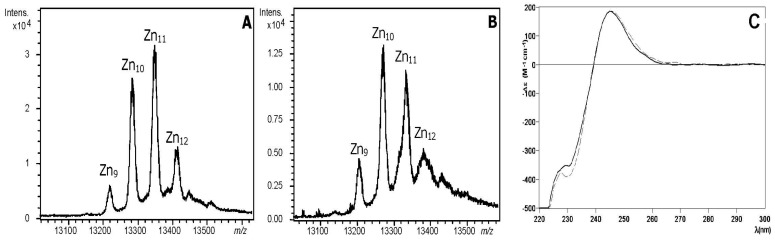
MS and circular dichroism (CD) spectra of Zn-BgMT: Deconvoluted ESI-MS spectra of the recombinant (**A**) Zn-BgwtMT and (**B**) Zn-BgKNMT, recorded at neutral (7.0) pH; (**C**) CD spectra of each Zn(II)-preparation: BgwtMT (solid line) and BgKNMT (dashed).

The recombinant production of BgwtMT in Zn-enriched media rendered a mixture of several metallated species (Figure 3, Table 1) with Zn_11_- and Zn_10_-BgwtMT being the most abundant ones (Figure 3A). Similarly, the production of the BgKNMT mutant form rendered the same mixture of metal-loaded species, even if the most intense peak correlates with Zn_10_-BgKNMT (Figure 3B). The CD spectra of both preparations (Figure 3C) are very similar (an exciton coupling band centered at approx. 240 nm, corresponding to the expected Zn-(SCys)_4_ binding chromophores), thus suggesting a similar folding of both proteins about the Zn(II) ions.

**Figure 4:**
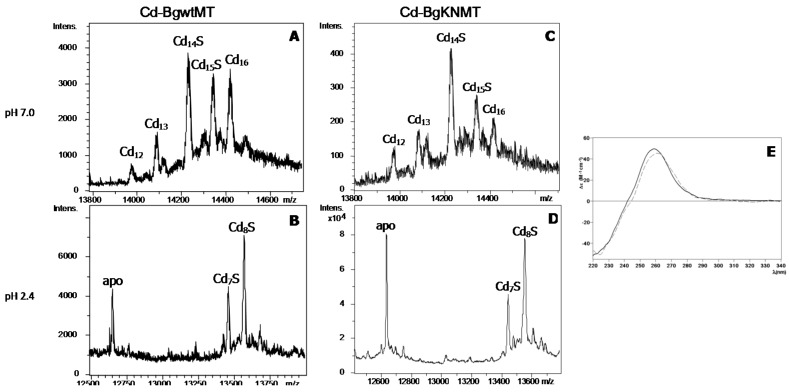
MS and CD spectra of Cd-BgMT: (**A**–**D**) Deconvoluted ESI-MS spectra of recombinant Cd-BgwtMT and Cd-BgKNMT, recorded at neutral (7.0) and acidic (2.4) pH; (**E**) CD spectra of each Cd(II) preparation: BgwtMT (solid line) and BgKNMT (dashed).

The ESI-MS analyses at neutral pH of the recombinant production of BgwtMT and BgKNMT in Cd-supplemented *E. coli* cultures (Figure 4, Table 1) revealed the formation of the same species in both preparations (with similar relative abundances), with the important presence of one major sulfide-containing species (Cd_14_S-BgMT) accompanied by minor amounts of Cd_15_-S, Cd_16_-, Cd_13_- and Cd_12_-MT. Interestingly, the mass spectra of both samples recorded at pH 2.4 revealed the remaining presence of Cd_7_S- and Cd_8_S-BgMT complexes coexisting with the apo-form. In every case, the decrease of pH down to 0.9 was necessary to completely remove all Cd(II) initially bound to proteins (data not shown). The CD spectra of the Cd-BgwtMT and Cd-BgKNMT preparations (Figure 4) display the contribution of both (1) a Gaussian band centered at ca. 250 nm, corresponding to the Cd-(SCys)_4_ chromophores and (2) a contribution of a further absorption at ca. 280 nm in agreement with the presence of Cd-S^2−^ chromophores in these samples. Furthermore, the congruence of the respective CD fingerprints confirmed equivalent folds of BgwtMT and BgKNMT when coordinating Cd(II) ions.

**Figure 5:**
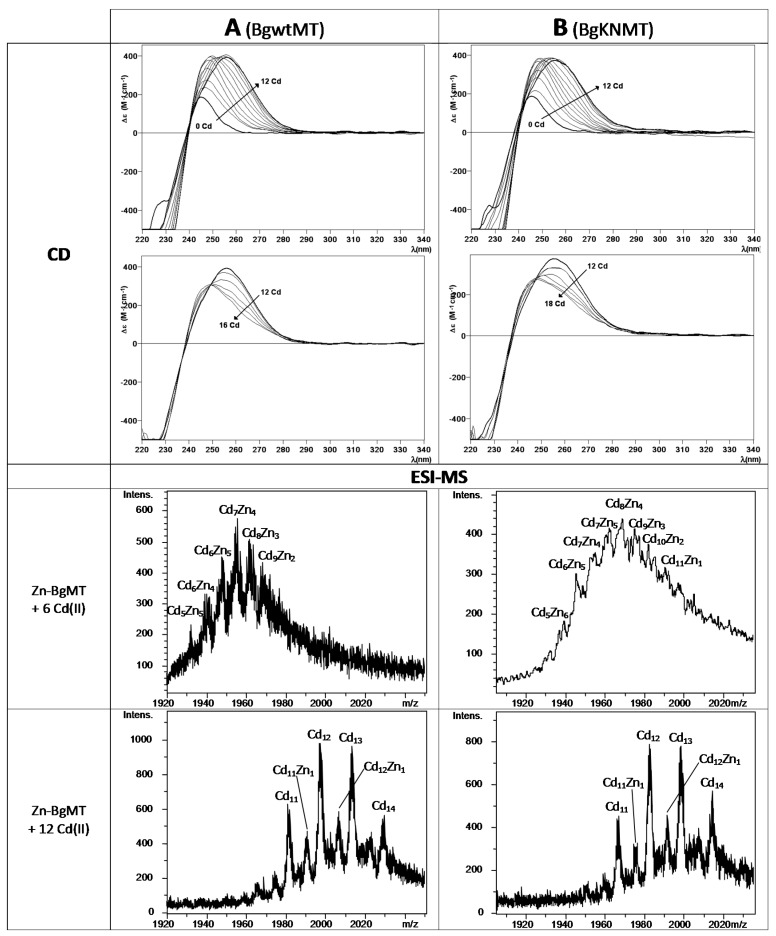
CD and ESI-MS spectra of Zn/Cd replacement of BgMT: Shown are spectra (at the +7 charge state) corresponding to the Zn(II)/Cd(II) replacement reaction of recombinant (**A**) Zn-BgwtMT and (**B**) Zn-BgKNMT, recorded at neutral pH. In both cases, a 5 µM solution of Zn-BgMT was titrated with up to 16–18 equivalents of CdCl_2_ at neutral pH.

In order to further study the capabilities of the BgwtMT and BgKNMT proteins for Cd(II) binding, the recombinant Zn-BgwtMT and Zn-BgKNMT preparations were titrated with Cd(II) and the Zn/Cd metal displacement reactions were followed in parallel by ESI-MS and CD spectroscopy (Figure 5). CD signals from both titrations proceeded very similarly: the addition of Cd(II) provoked a red shift of the maxima until 12 Cd(II) equivalents were added; further additions led to a decrease of the intensity suggesting unfolding of the formed clusters. These data are in agreement with the measured mass data. They show the subsequent replacement of the initial Zn(II) through formation of heteronuclear Zn, Cd-species, until the system is saturated after the addition 12 Cd(II) equivalents. Even when adding more Cd(II) the main species were still Cd_12_- and Cd_13_-BgMT. Other species (Cd_11_- and Cd_14_-BgMT as well as Cd_11_Zn_1_- and Cd_12_Zn_1_-BgMT) were also present to minor amounts.

### 2.3. Monovalent -Cu(I)- Binding Features of BgwtMT and BgKNMT

**Figure 6:**
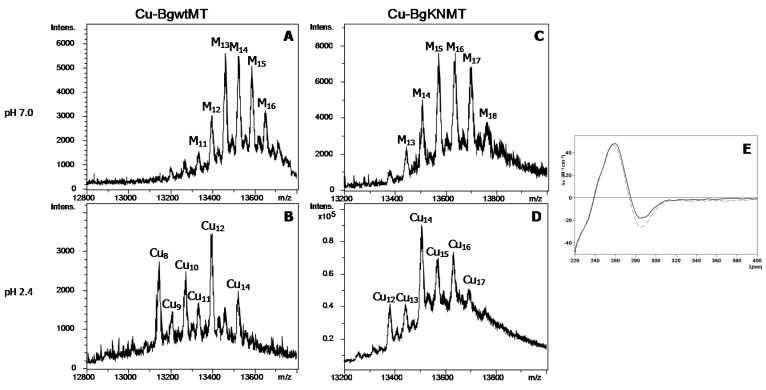
MS and CD spectra of Cu-BgMT: (**A**–**D**) Deconvoluted ESI-MS spectra of recombinant Cu-BgwtMT and Cu-BgKNMT, recorded at neutral (7.0) and acidic (2.4) pH. M denotes mixture of Zn and Cu (**E**) CD spectra of each Cu-preparation: BgwtMT (solid line) and BgKNMT (dashed).

The main differences between BgwtMT and BgKNMT were observed when producing them recombinantly in Cu-enriched media under standard or low aeration conditions (Figure 6). Although the two conventional types of Cu-supplemented productions [15] at standard (low intracellular copper content) or at low aeration (high intracellular copper content) conditions were assayed, several efforts to purify BgwtMT and BgKNMT from *E. coli* cultures grown at low oxygen conditions failed. Contrarily, both proteins could be isolated under normal aeration conditions, and were analyzed by ESI-MS and CD for comparison. Interestingly, both preparations exhibited a similar pattern: No stable species were observed, neither under the low aeration conditions nor under normal aeration conditions—both yielded a mixture of heterometallic Zn, Cu-species (Figure 6, Table 1).

However, while for BgwtMT the main species at neutral pH contains 13 metal ions (M_13_-BgwtMT, with M = Zn + Cu), and Cu_8_- and Cu_12_-BgwtMT at acidic pH, the natural BgKNMT mutant protein can bind more metal. This was observed at both pH values, with M_15_-_16_- and Cu_14_-BgKNMT being the main peaks at neutral and acidic pH, respectively (Figure 6C,D). The CD spectra were again very similar for both preparations (Figure 6E) and showed the typical absorbances at ca. 260 and 280 nm corresponding to Cu-loaded MTs. Even if considering that the metal-speciation was slightly different in both preparations, the coexistence of several heterometallic (Zn,Cu-BgwtMT and Zn,Cu-BgKNMT) species probably resulted in a similar folding of both proteins about the metal ions, always dominated by the characteristic signals of the more abundant Cu(I). Zn just revealed its presence as a faint shoulder at ca. 240 nm.

### 2.4. Lacking Metal-Binding Specificity is an Eminent Feature of Wild-Type and Allelic Biomphalaria Glabrata MTs

The fact that no single metallated species (with Zn^2+^, Cd^2+^ or even Cu^+^) could be obtained, neither in the recombinant productions, nor in the in vitro Zn/Cd replacement, indicates the lack of a metal specificity for both studied proteins. The absence of metal-binding specificity is thus considered as an inherent feature of BgwtMT and BgKNMT. Interestingly, the natural mutation K/N of the wild-type protein did not significantly affect its binding of divalent metal ions, and both proteins (BgwtMT and BgKNMT) behaved very similarly when binding Zn(II) and Cd(II) (similar speciation and very similar folding) (Figure 4). Contrarily, there were remarkable differences between the wild-type and the mutant variant with respect to binding of Cu(I). In fact, the replacement of one single amino acid (K by N) altered the binding properties of the protein by significantly increasing its Cu-binding capability, leading to species with a higher Cu(I) content than in the wild-type protein (Figure 6). This suggests that the replacement of K by N may have increased the Cu-thionein character of the unspecific BgwtMT protein, in agreement with previously reported data concerning the CaCdCuMT isoform of the *Cantareus aspersus* MTs [16]. Overall, however, this did not significantly change the unspecific binding character of both BgMT proteins.

The present findings are in contrast to the high metal-binding specificity of MTs from other gastropods, e.g., in case of the well characterized CdMT and CuMT isoforms of terrestrial snails from the Helicid family, including the Roman snail (*Helix pomatia*) [1,3] and the garden snail (*Cantareus aspersus*) [17], all belonging to the gastropod phylum of *Stylommatophora*. It is also true for the Cd-specific MT of the marine periwinkle (*Littorina littorea*), that belongs to the gastropod phylum of *Caenogastropoda* [4]. Altogether, our data indicate that metal-binding properties of gastropod MTs may vary in a lineage-specific manner.

### 2.5. Missing Metal Specificity of Recombinant BgMT Proteins Goes Hand in Hand with Lacking Upregulation of the BgMT Gene

**Figure 7:**
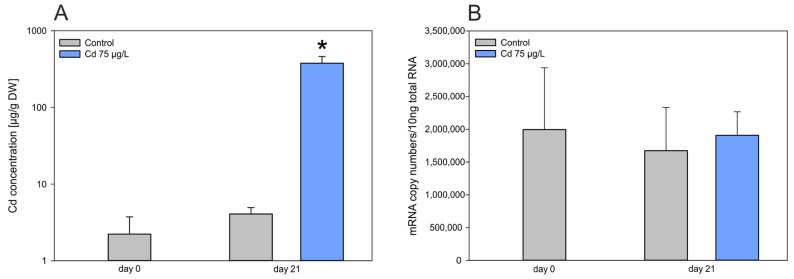
(**A**) Cd concentration in midgut gland tissue and qRT-PCR of mRNA after Cd exposure. (**A**) Cd concentration in midgut gland tissue of *Biomphalaria glabrata* on days 0 and day 21 of Cd exposure (75 µg/L); (* *p* < 0.001); the y-axis shows values in decadic logarithm; (**B**) Quantitative Real-Time PCR data of *BgwtMT* gene transcription on days 0 and 21 of Cd exposure (75 µg/L). Grey bars: control values; Blue bars: Cd exposure values.

While many genes of metal-specific *MT*s from other gastropod species can specifically be upregulated by exposure to their cognate metal ions [2,6], this was clearly not the case for the *MT* gene of *Biomphalaria glabrata (BgwtMT)*, at least upon exposure to Cd^2+^. While the metal itself was strongly accumulated in the midgut gland of Cd-exposed individuals with a concentration factor about 500 times above control levels (Figure 7A), there was no concomitant upregulation of the *MT* mRNA concentration at all (Figure 7B). Instead, it appeared that mRNA levels of the Bg*MT* in both untreated (control) and Cd-treated animals were already highly elevated, compared to transcription levels of other gastropod *MT* genes under control conditions. mRNA concentrations of the metal-specific *CdMT* genes of the terrestrial snails *Helix pomatia* and *Cantareus aspersus* under control conditions, for example [2,6], are about 8–15 times lower than the mRNA levels of the *BgMT* gene in untreated animals (Figure 7B). This strongly suggests that the *BgMT* gene and protein may function in a different manner than the metal-specific *MT* genes and proteins of *Helix pomatia* and *Cantareus aspersus* species. Considering the concomitance of the lack of metal specificity and the missing Cd-dependent inducibility of the *BgMT* gene, which is already highly expressed in controls, one obvious hypothesis is that the metal sequestration potential of BgMT may primarily be based on its metal replacement capacity rather than on a strong binding selectivity after metal-dependent induction and de novo synthesis. In fact, metal replacement reactions of the recombinant BgwtMT and BgKNMT proteins indicate that, at least in vitro, such replacement reactions (e.g., Cd^2+^ versus Zn^2+^) may take place without significantly impairing the protein integrity and structure (Figure 5).

### 2.6. The Biomphalaria glabrata MT in the Context of Other Hygrophila MTs: Deviant Primary Structures and Metal Stoichiometries

As indicated above, the unspecific metal-binding properties of recombinant BgMT and the lacking upregulation capacity of the corresponding gene grossly deviate from features of the rather metal-specific MT proteins and their responsive cognate genes of many other gastropod species. Indeed, *Biomphalaria glabrata* seems to share some of its deviating MT features with snail species belonging, just as *Biomphalaria glabrata* does, to the monophyletic gastropod clade of *Hygrophila* that comprises, according to the current phylogeny, all air-breathing (“pulmonate”) freshwater snails of the super-phylum of *Panpulmonata* [18]. While all so-far studied *Hygrophila* species seem to possess MTs [7,19], their cognate *MT* genes may only weakly or even hardly at all respond to heavy metal stress upon exposure. This was shown for Cd-exposed *Physa acuta* [9], metal-stressed *Lymnaea stagnalis* [20] or Cd-stressed *Biomphalaria glabrata* (this study). Instead, phytochelatin (PC) synthesis was recently demonstrated as a responsive reaction of *Lymnaea stagnalis* to Cd stress [8]. This suggests that apart from MTs, PC metal complexes may be formed in these aquatic snails in response to metal stress. It is not clear, however, if and how the PC system in these animals may interfere with the MT pool.

**Figure 8:**
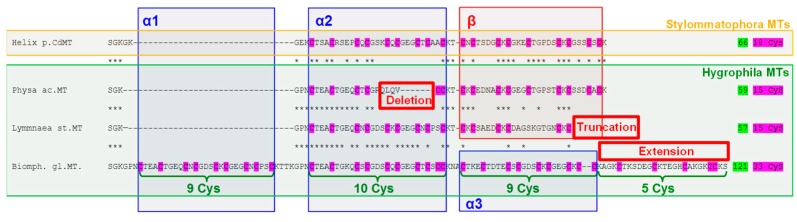
Sequence Alignment of sequences of various snail MTs: Alignment of *Helix pomatia* CdMT (Helix p.CdMT) (*Stylommatophora* MTs, **orange**-framed box) sequence with those of MTs of *Hygrophila* (*Hygrophila* MTs, **green**-framed box), including MTs of *Physa acuta* (Physa ac.MT), *Lymnaea stagnalis* MT (Lymnaea st.MT) and *Biomphalaria glabrata* MT (Biomph. gl.MT), showing the presumed domain organization with up to three α domains (α1, α2 and α3, **blue**-framed boxes) and one β domain (**red**-framed box). Conserved cysteine positions in the MT sequences are underlaid in pink. Also indicated in green letters are the numbers of Cys residues in every single domain of the *Biomphalaria gl.* MT, along with primary structure deviations (Deletion, Truncation, Extension) in *Hygrophila* MTs from the *Helix pomatia* CdMT model. Identical amino acid residues between the aligned sequences are indicated by stars. The respective amino acid chain lengths of the MT peptides are highlighted in **green** near their C-terminal ends. Also indicated next to the C-termini are the numbers of Cys residues in each MT sequence (underlaid in pink). GenBank Accession numbers of the shown sequences are as follows: *Helix pomatia* CdMT, ACN66299.1; *Physa acuta* MT, GU259686; *Lymnaea stagnalis* MT, KT253648; *Biomphalaria glabrata* MT KT697617.

Interestingly, a primary sequence alignment of the Cd-specific MT isoform of *Helix pomatia*, HpCdMT, with several MT sequences of *Hygrophila* species (Figure 8) shows that, in contrast to the former, the MT sequences of *Hygrophila* species (including *Physa acuta*, *Lymnaea stagnalis* and *Biomphalaria glabrata*), do not show a clear two-domain organization with a straight forward Cys:divalent metal-binding ratio of 9:3 per domain. Instead, the *Hygrophila* MT sequences seem to suffer from structural “degeneration” by deletion, truncation or extension of their primary sequences, often with deviations from the above-mentioned model of the 9:3 Cys:divalent metal ratio [21] (Figure 8). It is not known if and how all these structural deviations may influence the metal-binding behavior of the respective single proteins, but it may be assumed that overall, these structural “degenerations” from the classical gastropod MT model may contribute to impairment of their metal-specific binding properties.

## 3. Materials and Methods

### 3.1. Primary Structure of BglMT

The primary structure of the BglwtMT was elucidated by genome analysis within the *Biomphalaria glabrata* genome project (VectorBase, National Institutes of Health, Bethesda, MD, USA). The BglKNMT was obtained by screening for allelic variations and both sequences were experimentally verified by sequencing PCR-amplified and cloned individuals [13]. The sequences were submitted to the GenBank, and are available under the accession numbers KT697617 (BgwtMT) and KY963493 (BgKNMT).

### 3.2. Cloning and Heterologous Expression of Biomphalaria glabrata BgwtMT and BgKNMT

Synthetic cDNAs for the two allelic variants of the predicted *Biomphalaria glabarata* MTs were provided by Integrated DNA Technologies Company (Coralville, IA, USA). *Bam*HI and *Xho*I restriction sites and 6–7 additional 5’-nucleotides were added to the BgMT cDNA ends to facilitate the cloning processes. The synthetic cDNAs were PCR amplified with specific primers 5′-TTTTATT*GGATCC*ATGAGTGGCAAAG-3′ (forward) and 5′-ATTTTT*CTCGAG*TCAACTCTTAC-3′ (reverse), using Expand High Fidelity PCR system^®^ (Roche, Penzberg, Upper Bavaria, Germany). A 25-cycle amplification reaction was performed under the following conditions: 30 s at 94 °C, 30 s at 55 °C, and 45 s at 72 °C, in a 25 μL PCR mixture containing 25 ng of template DNA, 0.01 mM of each primer, and 0.0125 mM of each dNTP. The amplified products were analyzed on a 1% agarose gel stained with Gel red (Biotum Inc., Bay Area, CA, USA). The *BgMT* cDNAs were digested with *Bam*HI and *Xho*I enzymes, cloned into a *Bam*HI-*Xho*I digested pGEX-4T-1 vector (GE Healthcare, Chicago, IL, USA) with the DNA Ligation Kit 2.1^®^ (Takara Bio Inc., Shimogyo-ku, Kyoto, Japan), and transformed into *E. coli* Dh5α strain. Plasmid DNA was purified from bacteria using the GeneElute™ Plasmid Miniprep Kit (Sigma-Aldrich, St. Louis, MO, USA), screened for insert presence by digestion with ScaI enzyme, and sequenced using the BigDye^®^ Terminator v3.1 Cycle Sequencing Kit (Applied Biosystems, Waltham, MA, USA) in an ABIPRISM 310 automatic sequencer (Applied Biosystems). DNA from each recombinant *BgMT*-pGEX plasmid was used to transform *E. coli* BL21 strain, a protease deficient strain used for heterologous protein expression.

The recombinant expression of *BgMTs* was assayed by growing 3 mL of LB-25 mg/mL ampicillin medium inoculated with BgMT-producing *E. coli* BL21 strains. After growth overnight at 37 °C and 250 rpm, 0.3 mL of the culture was used to inoculate 3 mL of fresh medium and new cultures were grown for 2 h. The expression of the *BgMT*s was induced with 100 μM (final concentration) of isopropyl-β-d-thiogalactopyranoside (IPTG) for 3 h. After 30 min of induction, cultures were supplemented with 500 μM of CuSO_4_, 300 μM of CdCl_2_ or 300 μM of ZnCl_2_ (final concentrations), and allowed to grow for further 2.5 h for the synthesis of the respective metal complexes. Cells were harvested by centrifugation for 1 min at 13,000 rpm, and bacterial pellets were suspended by vortexing in 150 μL of Phosphate Buffered Saline (PBS1X) (140 mM NaCl, 2.7 mM KCl, 10.1 mM Na_2_HPO_4_, 1.8 mM Kh_2_PO_4_). Suspended cells were sonicated (Sonifier^®^ ultrasonic cell disruptor, Ferguson, MO, USA) at voltage 2 with 9 pulses of 0.6 s, and then centrifuged for 10 min at 12,500 rpm at 4 °C. Supernatant was recovered, and protein content was measured by a Bradford assay (Bio-Rad, Hercules, CA, USA ) in a Nanoquant infinite M200 microplate reader (Infinite M200 TECAN). Expression of both *BgMTs* was analyzed by sodium dodecyl sulfate polyacrylamide gel electrophoresis (SDS-PAGE) on 12.5% gels stained with Coomassie Blue.

### 3.3. Synthesis and Purification of Recombinant BgwtMT and BgKNMT

The BgMT metal complexes were biosynthesized in 5 L Erlenmeyer cultures of the corresponding transformed *E. coli* Bl21 cells grown in LB medium containing 100 mg/mL ampicillin supplemented with ZnCl_2_ (300 µM), CdCl_2_ (300 µM) or CuSO_4_ (500 µM). For copper cultures, two different aeration conditions were applied (250 rpm—standard aeration and 150 rpm—low aeration), of which only the standard aeration culture was successful. BgMT synthesis was induced with isopropyl-1-thio-β-d-galactopyranoside (IPTG) at a final concentration of 100 µM for 30 min. After adding the metal solution followed by a 2.5 h induction, cells were harvested by centrifugation. The protein containing pellets were re-suspended in 4 °C PBS 1× supplied with 0.5% (*v*/*v*) ß-mercaptoethanol, sonicated and centrifuged at 12,000× *g* for 40 min at 4 °C. Glutathione S-transferase (GST)-BgMT polypeptides were purified using batch affinity chromatography with glutathione sepharose (GE Healthcare, Buckinghamshire, UK). After incubation for one hour at room temperature applying constant agitation, the mix was washed in PBS three times. Argon was bubbled through all the washing steps following cell disruption to avert oxidation of the metal-BgMT complexes. Thrombin (GE Healthcare, Buckinghamshire, UK) (1 µL/mg of fusion protein) was added to the mixture and digestion was carried out overnight at 16 °C. This enabled separation of the GST fragment, which remained bound to the gel matrix, from the fusion protein. The eluted solution was applied to Centriprep low Concentrators (Amicon, Millipore, MA, USA) with a cut-off of 3 kDa and subsequently fractionated by means of fast protein liquid chromatography (FPLC), using a Superdex-75 column (GE Healthcare) equilibrated with 50 mM Tris-HCl, pH 7.0, and run at 1 mL/min. Fractions were collected and the protein content was analyzed by its absorbance at 254 nm. Samples containing BgMT were pooled and stored at −80 °C.

### 3.4. Zn(II)/Cd(II) Replacement Reactions in the Zn(II)-BgMT Proteins

Reactions of Zn(II) displacement by Cd(II) on the recombinant Zn-BgMT preparations were performed as described elsewhere [22]. This allowed the formation of the Cd-“in vitro complexes” by addition of several molar equivalents of Cd^2+^ from a standard solution to the corresponding Zn-BgMT preparation. These experiments were performed at constant pH 7.0 without the addition of any extra buffers, and under argon atmosphere.

### 3.5. Spectroscopic Analyses (ICP-AES, UV-Vis and CD) of the Metal Complexes Formed by the BgwtMT and BgKNMT Proteins

Determination of the sulfur and metal content of all the metal-BgMT samples was performed by Inductively Coupled Plasma Atomic Emission Spectroscopy (ICP-AES) in a Polyscan 61E (Thermo Jarrel Ash, Franklin, MA, USA) spectrometer by measuring S at 182.040 nm, Zn at 213.856 nm, Cd at 228.802, and Cu at 324.803 nm. This allowed determination of the protein concentration by considering that all S atoms were provided by the BgMT peptides. A Jasco spectropolarimeter (Model J-715, JASCO, Groß-Umstadt, Germany) interfaced to a computer (J700 software, JASCO, Groβ-Umstadt, Germany) was used for circular dichroism (CD) measurements. The electronic absorption measurements were performed in an HP-8453 Diode array UV-vis spectrophotometer (GIM, Ramsey, MN, USA) in 1-cm capped quartz cuvettes. In all spectroscopic measurements the dilution effects were corrected and processed using the GRAMS 32 software (Thermo Fisher Scientific, Waltham, MA, USA).

### 3.6. Electrospray Ionization Time-of-Flight Mass Spectrometry (ESI-TOF MS) of the Metal Complexes Obtained from the BgMT proteins

The *M*_W_ determinations by electrospray ionization time-of-flight mass spectrometry (ESI-TOF MS) were carried out in a Micro TOF-Q instrument (Bruker Daltonics, Bremen, Germany) interfaced with a Series 1200 HPLC Agilent pump and equipped with an autosampler, all of which were controlled by the Compass Software. ESI-L Low Concentration Tuning Mix (Agilent Technologies, Santa Clara, CA, USA) was used for calibration. The samples were analyzed using a 5:95 mixture of acetonitrile:ammonium acetate (15 mM, pH 7.0) or a 5:95 acetonitrile:formic acid mixture (at pH 2.4) as carrier buffers. The neutral pH buffer allowed to detect all the metallated species while the acidic conditions provoke the release of Zn(II) and Cd(II), but keep the Cu(I) ions bound to the proteins.

Experimental mass values were calculated as described in [23] and the error associated with the measurements resulted to be always smaller than 0.1%.

### 3.7. Experimental Set-Up for BgMT Gene Induction Studies

Individuals of *Biomphalaria glabrata* originated from a laboratory-grown culture at the Institute of Zoology in Innsbruck, where the snails were kept in freshwater aquarium tanks at 25 °C with a 12:12 h photoperiod. Snails were fed *ad libitum* with commercially available lettuce (*Lactuca sativa*) every third day.

Prior to the experiment, forty individuals of *Biomphalaria glabrata* were acclimatized for two weeks in reconstituted water (KCl 18 mg/L, MgSO_4_ 190 mg/L, NaHCO_3_ 98.5 mg/L, CaCl_2_ 450 mg/L and NaCl 430 mg/L in milliQ water). Afterwards, snails were separated into different tanks and a Cd exposure regime was applied by adding CdCl_2_ to a final Cd concentration of 75 µg/L. A control group of 20 individuals was kept in Cd-free reconstituted water as a reference. Resulting Cd concentrations in the water were as follows (mean ± standard deviation, *n* = 5): Control, 0.24 ± 0.14 µg/L; Cd exposure, 63 ± 7.6 µg/L. Throughout the experiment the snails were fed with lettuce *ad libitum*. Four snails of each group were sampled on day 0 and 21. All sampled individuals were dissected and the midgut gland tissue was used for RNA isolation and tissue Cd analysis as described below.

### 3.8. mRNA Isolation, Reverse Transcription and BgMT qRT-PCR

*Biomphalaria glabrata* individuals were dissected on an ice-cooled stainless steel plate and ~10 mg (fresh weight) of midgut gland tissue was used for RNA isolation. The remaining midgut gland tissue of each animal was processed further for Cd analysis as described below. Tissue samples were homogenized (Precellys, Bertin instruments, France) and total RNA was isolated with the RNeasy^®^Plant Mini Kit (Qiagen) applying on-column DNase 1 digestion (Qiagen). Quantification was achieved by means of the RiboGreen^®^RNA Quantification Kit from Molecular Probes (Invitrogen, Karlsruhe, Germany) on a VICTOR™X4 2030 Multilabel Reader (PerkinElmer, Waltham, MA, USA). 250 ng RNA were subjected to cDNA synthesis (Superscript^®^ IV Reverse Transcriptase, Invitrogen, Life Technologies, Waltham, MA, USA) in a 20 µL approach for subsequent Real-time Detection PCR.

Quantitative Real-time Detection PCR of *BgwtMT* cDNA was performed on a Quant studio 3 (Applied Biosystems, Thermo Fisher Scientific) using Power SYBR Green (Applied Biosystems). RT Primers were designed using the Primer Express 3.0 software (Applied Biosystems) and optimal primer concentrations were assessed with a primer-matrix followed by dissociation curves. The *BgMT* transcript with the defined amplicon length (107 bp) was amplified with the following primers and concentrations: *BgMT* sense, 900 nM; 5′-GCACTGACACAGAATGCAGTTG-3′ and *BgMT* antisense, 900 nM; 5′-TTTGCACCCTTCATCTGACTTAGT-3′ applying the following protocol of 40 cycles: denaturation at 95 °C for 15 s, annealing and extension combined at 60 °C for 60 s. Subsequently calibration curves from amplicons were generated to determine *C*_q_ values for copy number analysis (PCR efficiency ~96%) using the Thermo Fisher Cloud Software, Version 1.0 (Life Technologies Corporation, Waltham, MA, USA). The 10-µL PCR reaction contained 1 µL of cDNA and 1× Power SYBR Green PCR master mix, 1× U-BSA and sense and antisense primer.

### 3.9. Metal Analysis

Cd concentrations in the midgut gland tissues and the medium were assessed by flame atomic absorption spectrophotometry. Dry weight was determined after oven-drying the samples at 65 °C. Dry samples were pressure-digested in 2 mL tubes (Eppendorf, Hamburg, Germany) with a 1:1 mixture of nitric acid (Suprapure, Merck, Darmstadt, Germany) and deionized water in an aluminum oven covered with a heated lid at 69 °C until a clear solution was obtained. All samples were diluted to 2 mL with deionized water and Cd concentrations measured in the flame of an atomic absorption spectrophotometer (model Z-8200, Hitachi, Tokyo, Japan). Calibration was achieved using standard metal solutions in 1% nitric acid. Accuracy of metal measurements of the midgut gland was verified with certified standard reference material (TORT-2, Lobster Hepatopancreas Reference Material for Trace Metals; National Research Council Canada).

### 3.10. Statistical Methods

Data of qRT PCR and metal analysis were evaluated statistically by means of Sigma Plot 12.5. For normal-distributed data, the t-test was applied. For data failing equal distribution the Mann-Whitney rank sum test was used. Statistical significance was set at *p* ≤ 0.05.

## 4. Conclusions

The metal-binding capabilities of BgwtMT and its natural mutant BgKNMT, as studied in this work, revealed to be very similar in most of the investigated aspects. This is not of much surprise, as they differ by only one single mutation of a non-coordinating amino acid residue. To summarize, the two proteins share the following features: (1) They lack a clear metal-binding preference for any of the three metal ions assayed—Zn(II), Cd(II) or Cu(I). To this degree, this feature is clearly unprecedented in the world of *Gastropoda* MTs, even if some other MT isoforms of this sub-family are also characterized by a relatively small, but when compared to BgMT still significant, degree of metal-binding specificity [16,24]. (2) Consequently, the two MT variants of *Biomphalaria glabrata* (BgwtMT and BgKNMT) presented, in all the cases, mixtures of several differently metallated species. Accordingly we suspect that none of the uniquely metallated species presents a system significantly lower in energy compared to mixed-metal species. Hence, the metal-BgMT species found are almost equivalent in their Zn- and Cd-BgMT preparations. (3) The CD profiles of both protein variants are very similar when complexed to the same metal ion. (4) There are, however, slight differences in copper-binding between BgwtMT and BgKNMT: the Cu-BgKNMT preparations contain higher nuclearity (M = Zn + Cu) and higher copper content than the Cu-BgwtMT samples. (5) These observations can be explained if we consider the previously reported role of Lys and Asn residues for metal-binding preferences of snail MTs [16]: the replacement of a Lys residue (highly present in the MT isoforms with higher Zn- and/or Cd-thionein character) by an Asn residue (abundant in the so-called Cu-thioneins) reduces the Cd specificity in favor of Cu specificity. This increase of the Cu-thionein character is however rather limited, which we attribute to the high number of amino acids and Cys content (33 Cys residues among 126 amino acids) that reduces the influence of a single amino acid mutation. (6) The results from qRT-PCR do not show significant *BgMT* gene upregulation upon induction by Cd despite a strong accumulation of Cd in the midgut gland tissue. (7) This agrees well with the ESI-MS results that fail to show a clear metal-binding preference for BgMTs, while at the same time, indicate a high potential for metal replacement. This suggests that BgMT(s) in living cells may be more important for metal exchange (e.g., replacement of Zn^2+^ by Cd^2+^) through a constitutively abundant form, rather than for metal sequestration by an MT species that is highly upregulated in presence of a specific metal. (8) A comparison of the *Biomphalaria glabrata* MTs with the CdMT isoform of *Helix pomatia* and MTs of the gastropod clade of *Hygrophila* (to which *Biomphalaria glabrata* belongs) shows several deviations in primary structure from the classical domain organization of metal-specific gastropod MTs. These include primary sequence aberrations such as deletions, truncations and chain extensions, along with altering combinations of α and β domains. (9) Perhaps as a consequence, different ratios of Cys:divalent metal ions seem to be a common feature of *Hygrophila* MTs compared to the CdMT of *Helix pomatia*. (10) Thus, it is hypothesized that these structural “degenerations” of *Hygrophila* MTs from the classical gastropod MT model may contribute to the impairment of their metal-binding specificity and response properties. At the same time, this may clear the way for the activation of alternative detoxification strategies such as metal complexation by low molecular weight ligands.

## Figures and Tables

**Table 1:** Analytical characterization of the recombinant Zn-, Cd- and Cu-preparations of BgwtMT and BgKNMT. All data for the copper supplemented cultures correspond to normal aeration conditions, since no complexes could be recovered from low aerated cultures. Abbreviations: MT: Metallothionein, ICP-AES: Inductively coupled plasma atomic emission spectroscopy, ESI-MS: electrospray ionization mass spectrometry, Exp. MM: expected molecular mass, Calc. MM: calculated molecular mass.

Supplemented Metal	MT	ICP-AES ^a^	ESI-MS ^b^ pH 7.0	Exp. MM ^c^	Calc. MM ^d^	ESI-MS ^b^ pH 2.4	Exp. MM ^c^	Calc. MM ^d^
Zn	BgwtMT	9.8 Zn 0 Cd 0 Cu	Zn_9_- **Zn_10_-** **Zn_11_-** Zn_12_-	13,221 **13,286** **13,350** 13,414	13,222.8 **13,286.2** **13,349.6** 13,413.0	**apo-**	12,652	12,652.3
	BgKNMT	9.4 Zn 0 Cd 0 Cu	Zn_9_- **Zn_10_-** **Zn_11_-** Zn_12_-	13,207 **13,272** **13,336** 13,400	13,208.7 **13,272.1** **13,335.5** 13,398.9	**apo-**	12,639	12,638.2
Cd	BgwtMT	0 Zn 12.9 Cd 0 Cu	Cd_12_- Cd_13_- **Cd_14_S-** Cd_15_S- Cd_16_-	13,980 14,090 **14,229** 14,341 14,418	13,977.2 14,087.6 **14,230.0** 14,340.5 14,418.9	apo- Cd_7_S- **Cd_8_S-**	12,652 13,457 **13,567**	12,652.3 13,457.2 **13,567.6**
	BgKNMT	0 Zn 13.3 Cd 0 Cu	Cd_12_- Cd_13_- **Cd_14_S-** Cd_15_S- Cd_16_-	13,960 14,075 **14,215** 14,327 14,406	13,963.2 14,073.6 **14,216.0** 14,326.4 14,404.8	**apo-** Cd_7_S- **Cd_8_S-**	**12,638** 13,442 **13,553**	**12,638.2** 13,443.1 **13,553.5**
Cu	BgwtMT	3.7 Zn 0 Cd 9.5 Cu	M_11_-MT M_12_-MT **M_13_-MT** **M_14_-MT** M_15_-MT M_16_-MT	13,341 13,403 **13,467** **13,529** 13,588 13,653	13,340.4 13,402.9 **13,465.5** **13,528.0** 13,590.6 13,653.1	Cu_8_-MT Cu_9_-MT Cu_10_-MT Cu_11_-MT **Cu_12_-MT** Cu_13_-MT Cu_14_-MT	13,153 13,215 13,277 13,340 **13,403** 13,465 13,526	13,152.7 13,215.3 13,277.8 13,340.4 **13,402.9** 13,465.5 13,528.0
	BgKNMT	1.9 Zn 0 Cd 9.9 Cu	M_13_-MT M_14_-MT **M_15_-MT** **M_16_-MT** M_17_-MT M_18_-MT	13,452 13,514 **13,577** **13,639** 13,701 13,762	13,451.4 13,513.9 **13,576.5** **13,639.0** 13,701.6 13,764.1	Cu_12_-MT Cu_13_-MT **Cu_14_-MT** Cu_15_-MT Cu_16_-MT Cu_17_-MT	13,388 13,450 **13,514** 13,575 13,637 13,697	13,388.8 13,451.4 **13,513.9** 13,576.5 13,639.0 13,701.6

^a^ metal-to-peptide ratio calculated from S, Zn, Cd and Cu content (ICP-AES) data); ^b^ The stoichiometry of the metal-loaded complex was calculated from the mass difference between the holo- and the apo-peptides; Major species are highlighted in bold. M denotes mixtures of Zn and Cu; ^c^ Experimental molecular masses corresponding to the detected metal-complexes. The corresponding ESI-MS spectra are shown in Figure 2, Figure 3 and Figure 4 and Figure 6; ^d^ Theoretical molecular masses corresponding to the metal-complexes.
